# Supplementary material for: Lysosomal Machinery Drives Extracellular Acidification to Direct Non-apoptotic Cell Death
Source: Cell Rep. Author manuscript; Available in PMC 2019 Jul 8. (PMC6613820; doi:10.1016/j.celrep.2019.03.034)
Supplement: 1 [file NIHMS1526189-supplement-1.pdf]

Cell Reports, Volume *27*

Supplemental Information

Lysosomal Machinery Drives Extracellular Acidification to Direct Non-apoptotic Cell Death

Albert A. Mondragon, Alla Yalonetskaya, Anthony J. Ortega, Yuanhang Zhang, Oandy Naranjo, Johnny Elguero, Won-Suk Chung, and Kimberly McCall

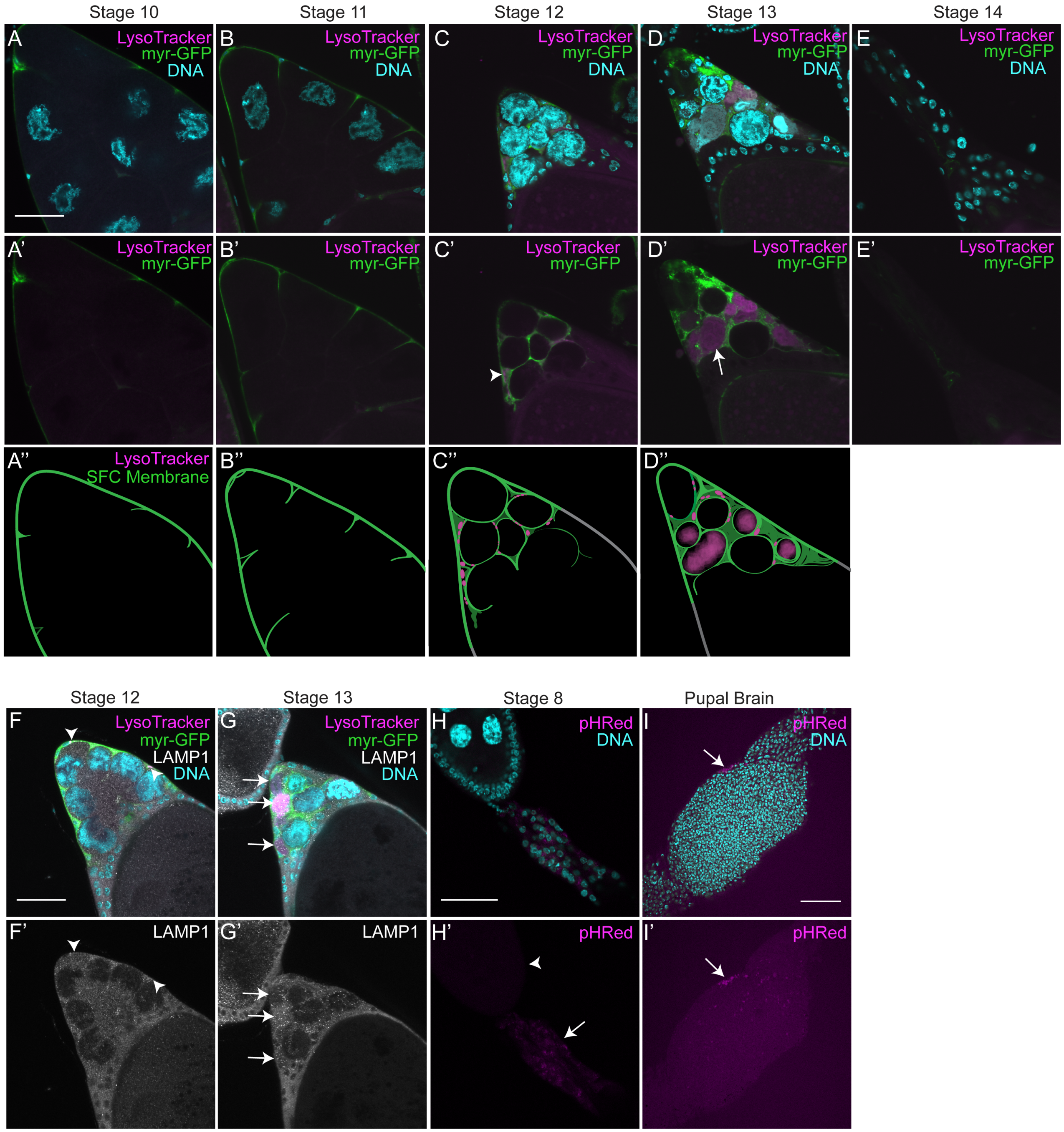

Supplemental Figure 1 – Analysis of pH sensitive markers, Related to Figure 1
(A-E') Comparison of LysoTracker staining and stretch follicle cell progression. *PG150(SFC)>myr-GFP* egg chambers labeled with LT (magenta). SFCs begin to surround NCs in stage 11. LT puncta accumulate around NCs within SFCs beginning at stage 12 (arrowhead). NCs become acidified at stage 13 (arrow). LT staining and SFC membranes are no longer detected in stage 14. **(A''-D'')** Schematic showing patterns of SFC membranes compared to LysoTracker. (**F-G'**) Comparison of LysoTracker with lysosomal marker LAMP-1. *PG150(SFC)>myr-GFP* egg chambers labeled with LT (magenta), LAMP-1 (White), and DAPI (cyan). (**H-I'**) Demonstration of pHRed as an engulfment marker. (**H-H'**) *NGT;nanos (germline)>pHRed* egg chambers labeled with DAPI (cyan). Healthy egg chamber (arrowhead) next to pHRed positive dying stage 8 egg chamber (arrow). (**I-I'**) *elav* (neuronal)>*pHRed* pupal brain labeled with DAPI (cyan). pHRed vesicles associate with regions of cell death in a pupal brain (arrows). Scale bars = 50 µm.

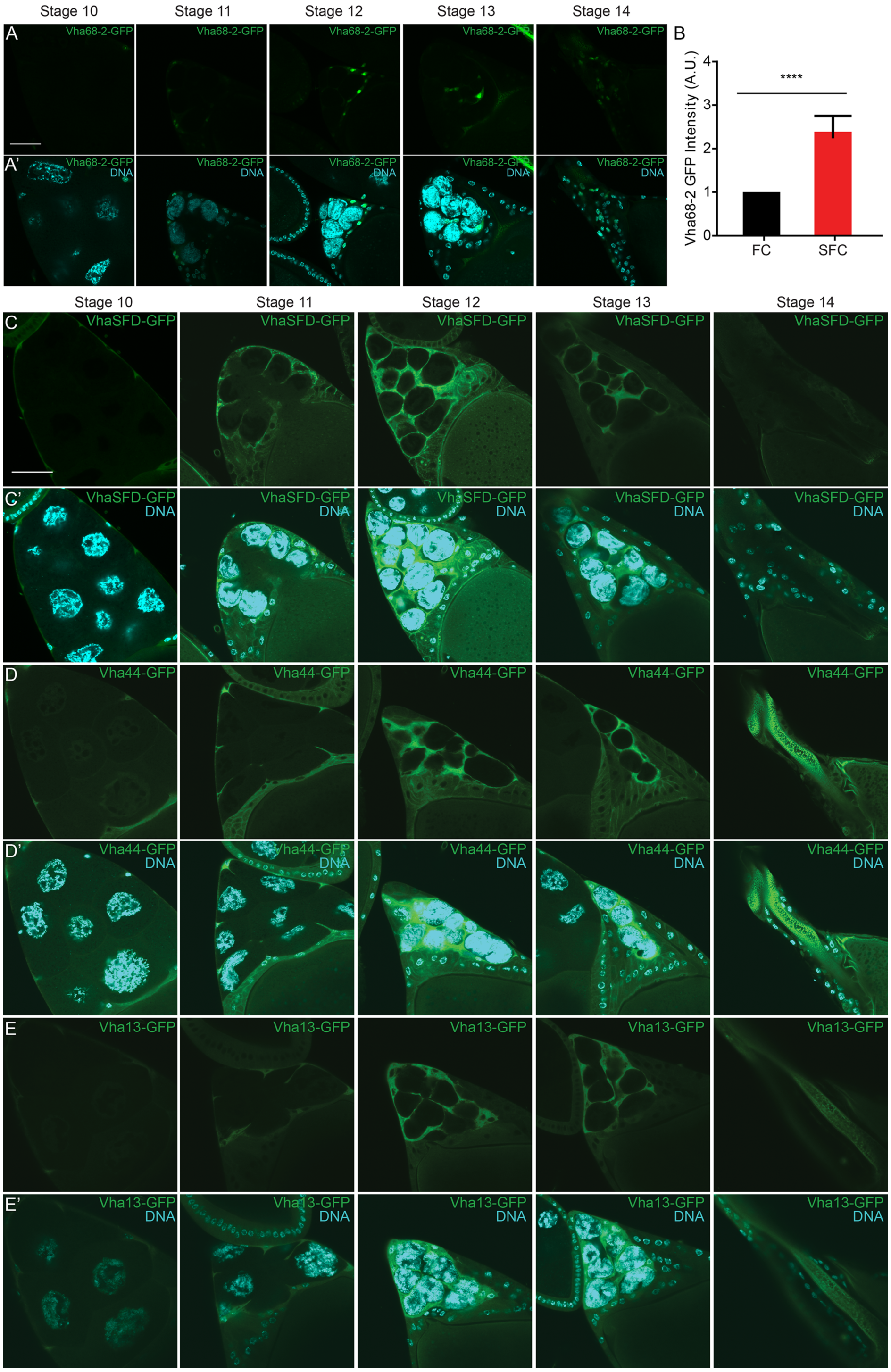

Supplemental Figure 2 – Vha68-2 enrichment in stretch follicle cells, and VhaSFD, Vha44, and Vha13 enrichment and localization in stretch follicle cells, Related to Figure 3

(A-A') *Vha68-2-GFP* enhancer trap (green) stage 10-14 egg chambers with DAPI (cyan). (**B**) Vha68-2 GFP intensity (arbitrary units) in follicle cells (n=33) and stretch follicle cells (n=29). Two-tailed students t-test, data are mean +/- SEM. **** $P \leq 0.0001$. Scale bar = 50 µm. (**C-C'**) *VhaSFD-GFP* protein trap (green) stage 10-14 egg chambers with DAPI (cyan). (**D-D'**) *Vha44-GFP* protein trap (green) stage 10-14 egg chambers with DAPI (cyan). (**E-E'**) *Vha13-GFP* protein trap (green) stage 10-14 egg chambers with DAPI (cyan). Scale bar = 50 µm.

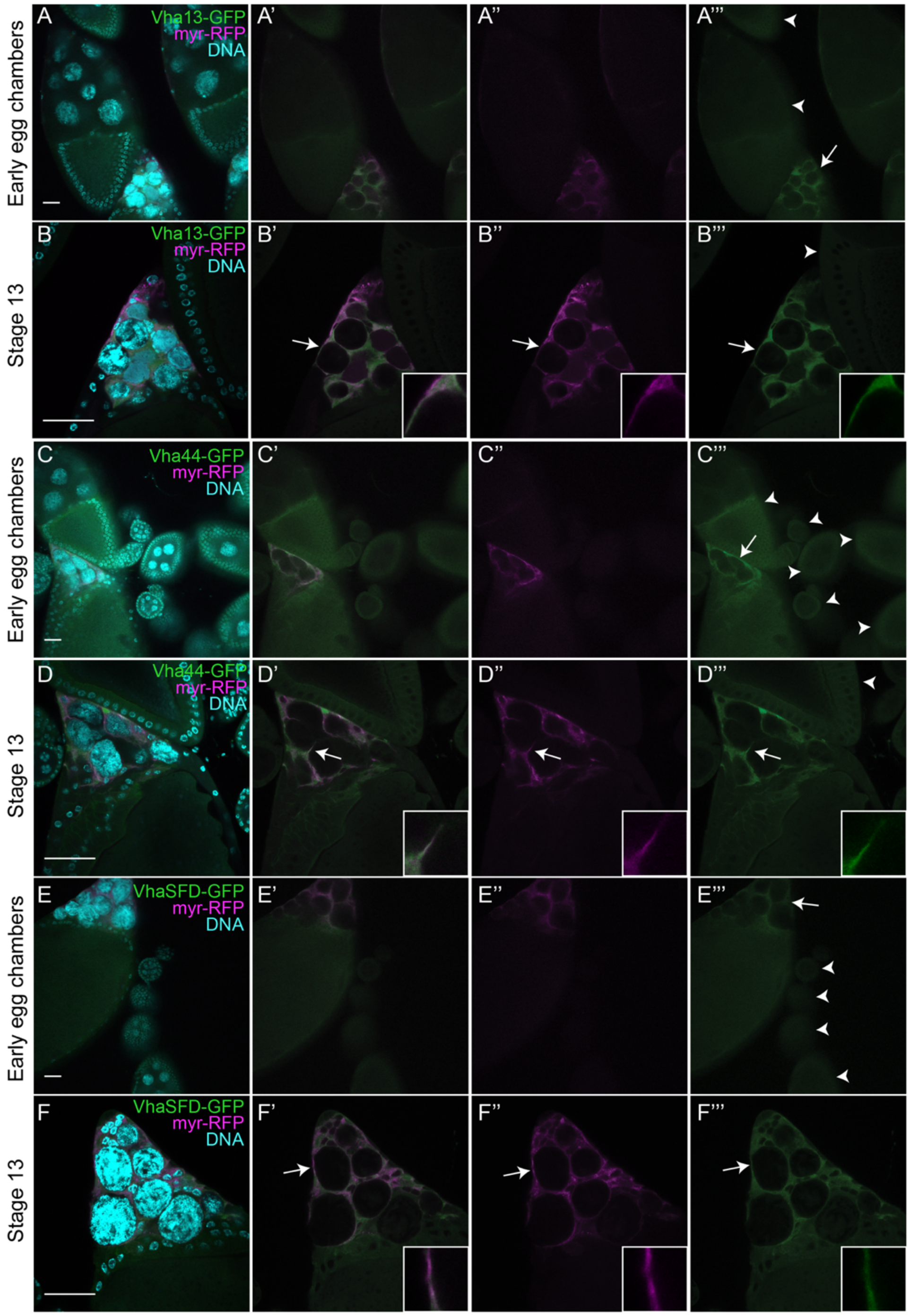

Supplemental Figure 3 – V-ATPases localize to the plasma membrane of stretch follicle cells, Related to Figure 3

(A-F''') Egg chambers expressing *PG150(SFC)>myr-RFP* (magenta) and the indicated V-ATPase subunit protein traps (green) stained with DAPI (cyan). (**A-A''', C-C''', E-E'''**) are lower magnification images with early stage egg chambers (arrowheads) compared to stage 13 egg chambers (arrow). The V-ATPase GFP protein traps are enriched in the SFCs of stage 13 egg chambers. (**B-B''', D-D''', F-F'''**) are higher magnification images of the stage 13 egg chambers. The V-ATPase GFP protein traps are localized to the stretch follicle cell plasma membrane (arrow). Insets show higher magnification of the overlap of V-ATPase GFP (green) and stretch follicle cell plasma membrane (magenta). Scale bars = 50 µm.

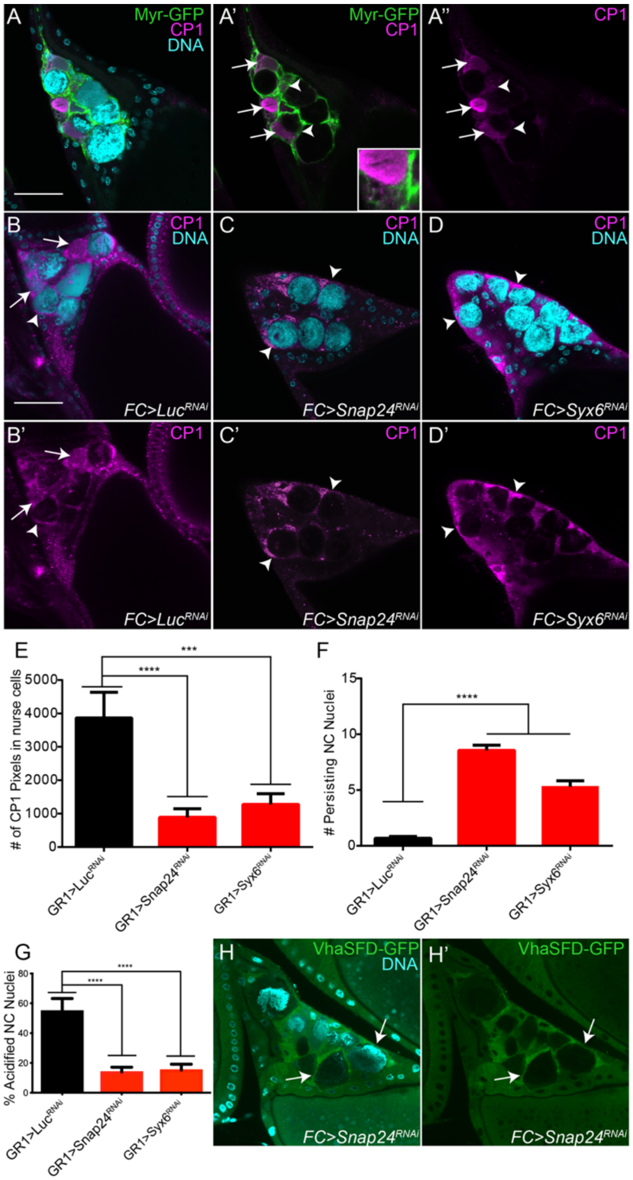

Supplemental Figure 4 – Snap24 and Syx6 are required for nurse cell removal and CP1 release, Related to Figure 4

(A-A'') *PG150(SFC)>myr-GFP* (green) stage 13 egg chamber labeled with anti-CP1 (magenta) and DAPI (cyan). CP1 is present in nurse cells (arrows) and stretch follicle cells (arrowheads). Inset shows CP1 localization inside nurse cell and stretch follicle cell. (**B-D'**) Stage 13 egg chambers from RNAi knockdowns labeled with anti-CP1 (magenta) and DAPI (cyan). In the control (B-B') CP1 surrounds some of the nurse cells (arrowheads) and is in other nurse cells (arrows). In the *Snap24* and *Syx6* knockdowns, CP1 is predominantly in the SFCs. **E,** Quantification of CP1 pixels which overlap nurse cell nuclei. The amount of CP1 in nurse cells is lower in the *Snap24* and *Syx6* knockdowns compared to the *Luc* control. Sample size: *GR1 (FC)>Luc^{RNAi}*, n = 20; *GR1(FC)>Snap24^{RNAi}*, n= 22; *GR1(FC)>Syx6^{RNAi}*, n = 24. Two-tailed students t-test, data are mean +/- SEM. **** P<0.0001 and *** P<0.005. (**F**) Quantification of persisting nurse cell nuclei in stage 14 egg chambers. Sample size: *GR1 (FC)>Luc^{RNAi}*, n = 84; *GR1(FC)>Snap24^{RNAi}*, n= 41; *GR1(FC)>Syx6^{RNAi}*, n = 51. Two-tailed students t-test, data are mean +/- SEM. **** P<0.0001. (**G**) Quantification of acidification of NCs in stage 13 egg chambers. Sample size: *GR1 (FC)>Luc^{RNAi}*, n = 10 ; *GR1(FC)>Snap24^{RNAi}*, n= 32 ; *GR1(FC)>Syx6^{RNAi}*, n = 22 . Two-tailed students t-test, data are mean +/- SEM. **** P<0.0001. (**H-H'**) *VhaSFD-GFP* protein trap (green, arrows) with RNAi knockdown of *Snap24* and labeled with DAPI (cyan). Scale bars = 50 μm.
