# Supplementary material for: Lysosomal Machinery Drives Extracellular Acidification to Direct Non-apoptotic Cell Death
Source: Cell Rep. Author manuscript; Available in PMC 2019 Jul 8. (PMC6613820; doi:10.1016/j.celrep.2019.03.034)
Supplement: 3 [file NIHMS1526189-supplement-3.pdf]

Report

Cell Reports

Lysosomal Machinery Drives Extracellular Acidification to Direct Non-apoptotic Cell Death

Graphical Abstract

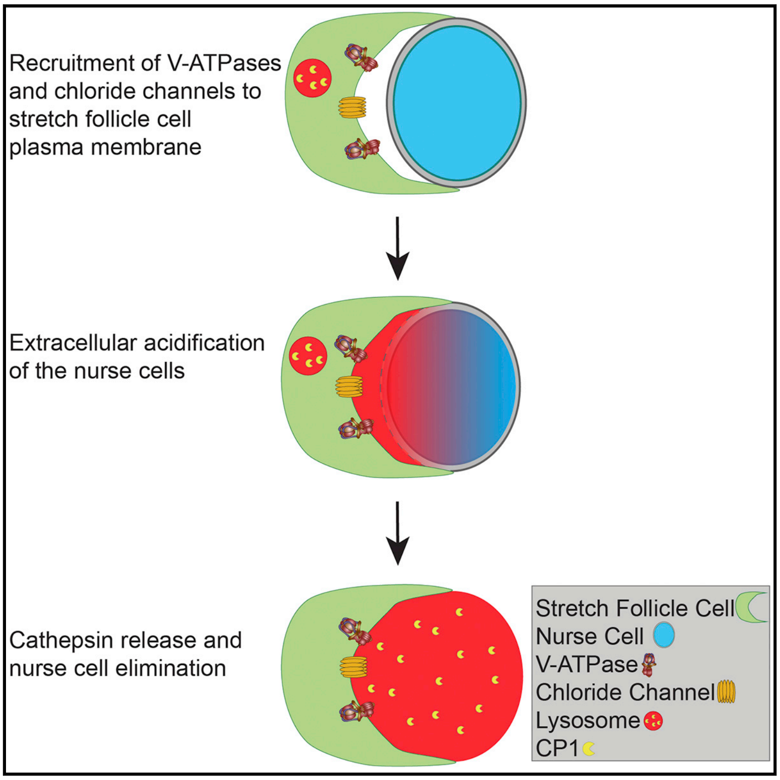

Authors

Albert A. Mondragon, Alla Yalonetskaya, Anthony J. Ortega, ..., Johnny Elguero, Won-Suk Chung, Kimberly McCall

Correspondence

kmccall@bu.edu

In Brief

Mondragon et al. show that V-ATPase proton pumps localize to the plasma membrane of follicle cells and promote extracellular acidification to eliminate adjacent nurse cells in the *Drosophila* ovary. The follicle cells subsequently release cathepsins by exocytosis into the nurse cells to promote their final degradation.

Highlights

- Lysosome-associated genes are required in follicle cells for nurse cell death
- V-ATPases localize to the plasma membrane of follicle cells to acidify nurse cells
- Cathepsin L is released by follicle cells and promotes nurse cell DNA degradation

Mondragon et al., 2019, Cell Reports *27*, 11–19
April 2, 2019 © 2019 The Author(s).
https://doi.org/10.1016/j.celrep.2019.03.034

Cell Reports
Report
OPEN ACCESS
CellPress

Lysosomal Machinery Drives Extracellular Acidification to Direct Non-apoptotic Cell Death

Albert A. Mondragon,[1,2] Alla Yalonetskaya,[1] Anthony J. Ortega,[1] Yuanhang Zhang,[1] Oandy Naranjo,[1] Johnny Elguero,[1] Won-Suk Chung,[3] and Kimberly McCall[1,4,*]
[1]Department of Biology, Boston University, 5 Cummington Mall, Boston, MA 02215, USA
[2]Program in Molecular Biology, Cell Biology, and Biochemistry, Boston University, Boston, MA 02215, USA
[3]Department of Biological Sciences, KAIST, Daejeon, South Korea
[4]Lead Contact
*Correspondence: kmccall@bu.edu
https://doi.org/10.1016/j.celrep.2019.03.034

SUMMARY

Cell death is a fundamental aspect of development, homeostasis, and disease; yet, our understanding of non-apoptotic forms of cell death is limited. One such form is phagoptosis, in which one cell utilizes phagocytosis machinery to kill another cell that would otherwise continue living. We have previously identified a non-autonomous requirement of phagocytosis machinery for the developmental programmed cell death of germline nurse cells in the *Drosophila* ovary; however, the precise mechanism of death remained elusive. Here, we show that lysosomal machinery acting in epithelial follicle cells is used to non-autonomously induce the death of nearby germline cells. Stretch follicle cells recruit V-ATPases and chloride channels to their plasma membrane to extracellularly acidify the germline and release cathepsins that destroy the nurse cells. Our results reveal a role for lysosomal machinery acting at the plasma membrane to cause the death of neighboring cells, providing insight into mechanisms driving non-autonomous cell death.

INTRODUCTION

Programmed cell death is essential in the development of an organism for elimination of dangerous cells and to maintain homeostasis (Jacobson et al., 1997). Apoptosis is the most heavily studied type of cell death (Galluzzi et al., 2015); however, it has recently been proposed that apoptosis may not be the most prevalent form of cell death in vertebrate development (Kutscher and Shaham, 2017). Work on non-apoptotic forms of cell death over the last decade has culminated in five proposed classifications of cell death: apoptotic, autophagic, necrotic, non-cell autonomous, and atypical cell death (Martins et al., 2017).

Entosis and phagoptosis are both types of cellular cannibalism that fall within the non-cell autonomous classification of cell death modalities. In entosis, internalized cells form adheren junctions and invade the neighboring cell, bypassing any requirement for conventional phagocytosis machinery (Overholtzer et al., 2007). In contrast, phagoptosis utilizes phagocytosis machinery to drive the death of a nearby cell that would otherwise continue living (Brown and Neher, 2012, 2014). Phagoptosis has been suggested to promote physiological cell deaths, such as neuronal loss associated with stroke (Lana et al., 2017; Neher et al., 2013), Parkinson's disease (Barcia et al., 2012), amyotrophic lateral sclerosis (ALS) (Liu et al., 2012), and retinitis pigmentosa (Zabel et al., 2016; Zhao et al., 2015).

Developmental germ cell death is a common phenomenon during oogenesis throughout metazoans (Peterson et al., 2015; Tilly, 2001). The *Drosophila* ovary provides a particularly powerful *in vivo* model for non-apoptotic germ cell death, given the large size of the cells, genetic tools, and reproducibility of cell death. Each *Drosophila* ovary is comprised of hundreds of developing egg chambers, each composed of 15 germline nurse cells (NCs) connected to a single oocyte, surrounded by a layer of follicle cells (FCs) (King, 1970). At the end of oogenesis, the NCs dump their cytoplasmic contents into the oocyte, become surrounded by a subset of FCs called stretch follicle cells (SFCs) (Duhart et al., 2017), and are eliminated without the requirement of apoptosis or autophagy genes (Peterson and McCall, 2013). We have previously found that the SFCs require phagocytic machinery to eliminate the NCs, demonstrating that the NCs die by phagoptosis (Santoso et al., 2018; Timmons et al., 2016, 2017); however, our understanding of how NC elimination is carried out remained limited.

Our previous studies revealed a role for lysosomal genes in NC death; however, the exact contribution of lysosomal genes was unknown (Bass et al., 2009; Timmons et al., 2016). Lysosomes have diverse functions: they are responsible for the degradation of materials in endocytosis or autophagy, repairing the plasma membrane through lysosome secretion, and metabolic signaling (Settembre et al., 2013). Lysosomes have also been linked to cell death. For example, in entosis, lysosomes act as the final executioner as they fuse with the internalized cell. Lysosomes contain over 50 acid hydrolases that are involved in degradation (Lübke et al., 2009). Of particular interest are cathepsins, which are lysosomal proteases that require acidic conditions to be proteolytically active. Cathepsins can also be secreted by some specialized cells, such as osteoclasts, to degrade bone and by cancer cells to facilitate invasion (Baron et al., 1988; Rozhin et al., 1994).

This is an open access article under the CC BY license (http://creativecommons.org/licenses/by/4.0/).

Vacuolar-type H$^+$-ATPases (V-ATPases) are a vital component of the lysosome that maintain the acidic pH by pumping protons into the lumen. V-ATPases are composed of a transmembrane complex and a cytoplasmic complex that together hydrolyze ATP to pump protons across a membrane (Cotter et al., 2015). V-ATPases have 14 subunits, encoded by 33 genes in *Drosophila*, with many of the genes having tissue-specific expression (Allan et al., 2005). V-ATPases are well known for their roles in acidification of lysosomes; however, V-ATPases also play an important role at the plasma membrane in specific cell types in humans, such as osteoclasts for bone resorption (Qin et al., 2012), intercalated cells in the kidney to regulate systemic pH (Brown et al., 2009), clear cells in the testis to maintain acidic luminal fluid (Shum et al., 2009), and cancer cells to acidify the extracellular matrix to facilitate invasion (Stransky et al., 2016).

Here, we report the essential role of lysosome-associated genes in NC death. Specifically, we show a non-autonomous role for V-ATPases and cathepsins in NC acidification and elimination. V-ATPases are enriched and recruited to the plasma membrane of the SFCs to extracellularly acidify the NCs, and cathepsins are released from the SFCs to drive NC degradation in a manner resembling osteoclast degradation of bone. Altogether, this work characterizes a new role for V-ATPases and cathepsins acting at the plasma membrane to drive the death of a neighboring cell.

RESULTS

Nurse cells Are Acidified Extracellularly

Fifteen NCs are connected to each developing oocyte from the earliest stages of oogenesis through ring canals formed by incomplete cytokinesis (Spradling, 1993). The NCs support the growth and development of the oocyte throughout oogenesis by delivery of organelles, proteins, and RNA to the oocyte. Near the end of oogenesis, the NCs begin to show signs of cell death beginning at stage 10, with distinct changes, including cytoskeletal rearrangements, the leakage of nuclear material, and nuclear remodeling seen by transmission electron microscopy (TEM) (Cooley et al., 1992; Guild et al., 1997). During stage 11, the NCs rapidly transfer their cytoplasm into the oocyte (Spradling, 1993). The nearby SFCs surround the NCs by stage 12 (Duhart et al., 2017) and are required for multiple cell death events in the NCs, including cytoskeletal rearrangements and cytoplasm transfer (Timmons et al., 2016). During stages 12 and 13, the NCs become acidified and DNA is fragmented (Bass et al., 2009; Foley and Cooley, 1998). The NCs are subsequently degraded by stage 14, leaving the fully intact mature oocyte.

One of the most unusual cell death events of the NCs is their complete acidification (Bass et al., 2009; Timmons et al., 2016). Previously, we had determined that acidic organelles were detected in SFCs prior to the acidification of the NCs, but it was unclear how the NCs became acidified. To investigate how NCs become acidified, we recorded time-lapse images of stage 13 egg chambers, the developmental stage when NC acidification occurs (Figures 1A–1E'). SFC membranes were visualized using an SFC-specific GAL4 (see STAR Methods) to drive expression of a membrane-tethered (myristoylated) GFP. To detect acidification of the NCs, egg chambers were labeled with LysoTracker (LT), an acidotropic dye (Timmons et al., 2013). During stage 13, mobile acidic vesicles in the SFCs were observed surrounding NCs prior to their acidification. Fixed tissue staining also demonstrated an accumulation of acidic vesicles in SFCs before the acidification of NCs (Figures 1F–1G' and S1A–S1E) (Timmons et al., 2016). The acidic vesicles and acidified NCs exhibited a different pattern than lysosomes detected by LAMP1 staining (Figures S1F–S1G). Live imaging over the course of an hour showed the dynamic activity and accumulation of LT vesicles in the SFCs before NC acidification (Video S1).

To further understand the process of NC acidification, we generated flies with a membrane-bound pH detector, pHRed-CAAX (Figure 1H). pHRed is a genetically encoded pH sensor (Tantama et al., 2011) modified with a CAAX motif that localizes pHRed to the cytoplasmic side of the plasma membrane (Hancock et al., 1991). To confirm that pHRed served as an engulfment detector, we expressed it in neurons and NCs and monitored its fluorescence following induction of apoptosis. In both cases, pHRed was not detected in healthy cells but was detected as punctate staining adjacent to dying cells, suggesting that the dying cell material was taken up and acidified within phagosomes (Figures S1H–S1I'). We next characterized pHRed in late-stage NCs to determine how NCs were acidified during developmental cell death. Unlike the labeling from engulfed apoptotic cells, pHRed was first detected along the NC membrane adjacent to SFCs, followed by pHRed labeling of entire NC remnants (Figures 1I–1J'). These two distinct phases of pHRed detection indicate that acidification initiates when the NC membrane is intact and progresses as the NC membrane is broken down and dispersed throughout the cell. The initial acidification of the membrane suggests that the NCs are acidified extracellularly by the FCs and subsequently degraded.

V-ATPases Are Required for Acidification and Clearance of NCs while Cathepsins Are Only Required for Clearance of NCs

Previously, we discovered that lysosomal trafficking genes were required non-autonomously in the FCs for NC acidification (Timmons et al., 2016). To further understand the role of lysosomes in NC elimination, we screened additional lysosome-associated genes using RNAi to knock down selected genes in the FCs and determine the effect on the NCs. The screen revealed a major requirement for V-ATPases and cathepsins. Although lysosomes are an essential component of phagosome maturation, little is known about the potential requirement of lysosomal machinery for cell death. Given the unusual acidification of NCs, we tested whether these lysosomal components were required for NC acidification and clearance. In normal development, NCs are asynchronously acidified and cleared between stages 13 and 14; quantification revealed that 50.5% of NCs were acidified in stage 13 and 91.9% were cleared by stage 14 (Figures 2A, 2A', 2E, and 2F). Knock down of V-ATPase subunits Vha100-2 or Vha16-1 in FCs significantly reduced the acidification of NCs in stage 13 egg chambers to 6.6% and 5.6% respectively (Figures 2B, 2C, and 2E). In contrast, no acidification defect was observed when CP1, the *Drosophila* ortholog of cathepsin L (Tryselius and Hultmark, 1997), was knocked down (Figures 2D

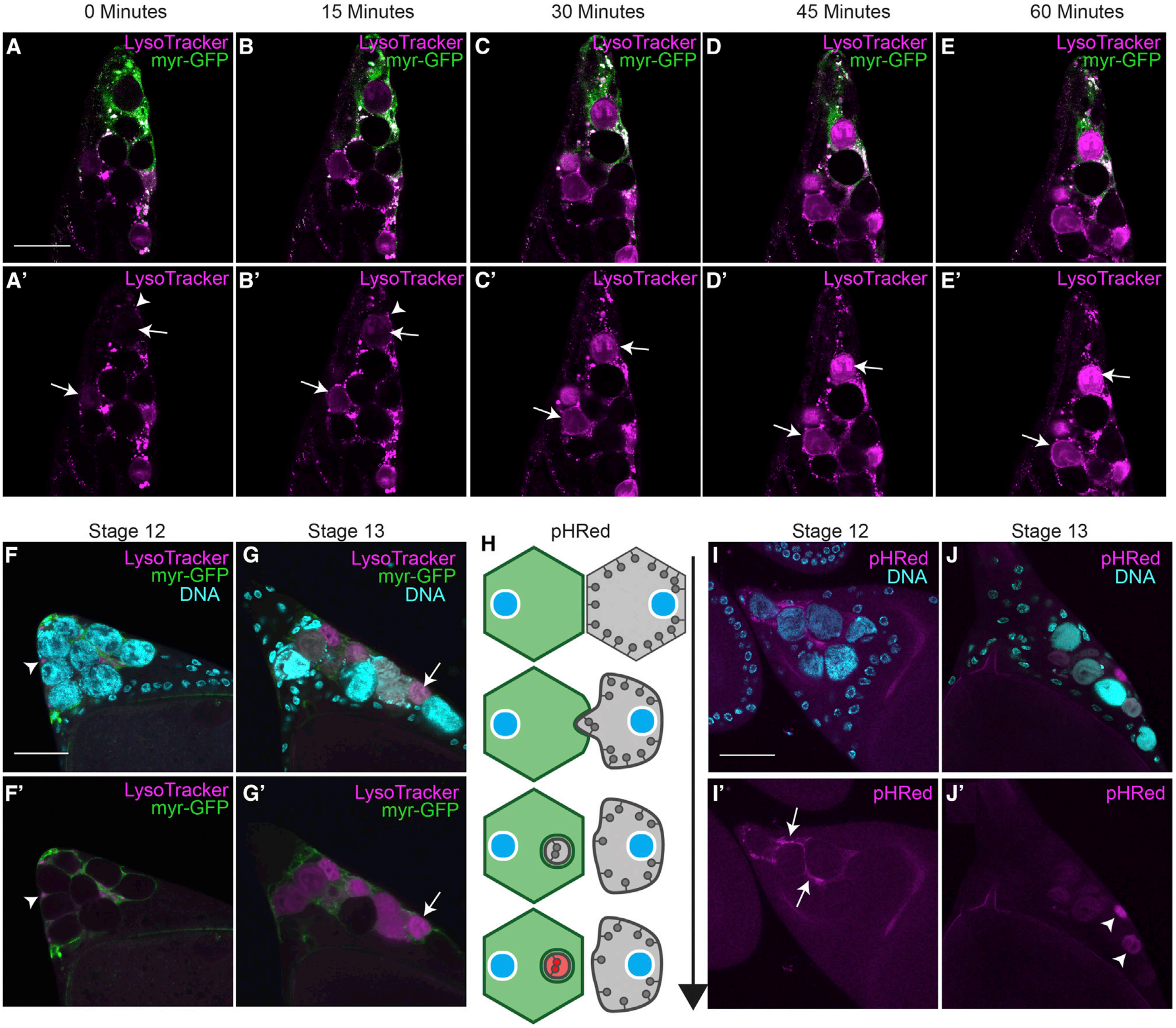

Figure 1. Nurse Cells Are Surrounded by Stretch Follicle Cells and Acidified

(A–E) Time lapse images of stretch follicle cell (SFC) > *myr-GFP* (green) stage 13 egg chamber labeled with LysoTracker (LT, magenta).

(A'–E') The same images with the LT channel only. LT puncta accumulate around nurse cells (NCs) within SFCs (arrowheads in A' and B') as NCs become acidified (arrows in A'–E') over 60 min.

(F and G) *SFC>myr-GFP* stage 12 (F) and stage 13 (G) egg chambers stained with DAPI (cyan) and LT (magenta).

(F' and G') The same egg chambers showing only the GFP and LT channels.

(F and F') LT puncta accumulate around NCs in stage 12 (arrowhead).

(G and G') NCs are acidified in stage 13 (arrow).

(H) Diagram of pHRed as an acidification detector, adapted from Fishilevich et al., (2010). pHRed is targeted to the cytoplasmic side of the plasma membrane and fluoresces red upon acidification.

(I and J) Germline > *pHRed* egg chambers stained with DAPI (cyan).

(I) Acidification of NC membrane detected by pHRed in stage 12.

(J) NCs in stage 13 are pHRed positive. Scale bars, 50 μm.

and 2E). Knock down of either of the V-ATPase subunits or CP1 resulted in persisting NCs (Figures 2B'–2D' and 2F). These findings suggest a two-step process where V-ATPase activity is required in the SFCs to first acidify the NCs and, subsequently, the SFCs utilize CP1 for NC degradation.

V-ATPases Are Enriched in Stretch Follicle Cells and Localize to the Plasma Membrane

To visualize V-ATPase expression in the ovary, we examined reporter lines for several of the subunits. We first examined a Vha68-2 (subunit A) enhancer trap that expresses nuclear GFP

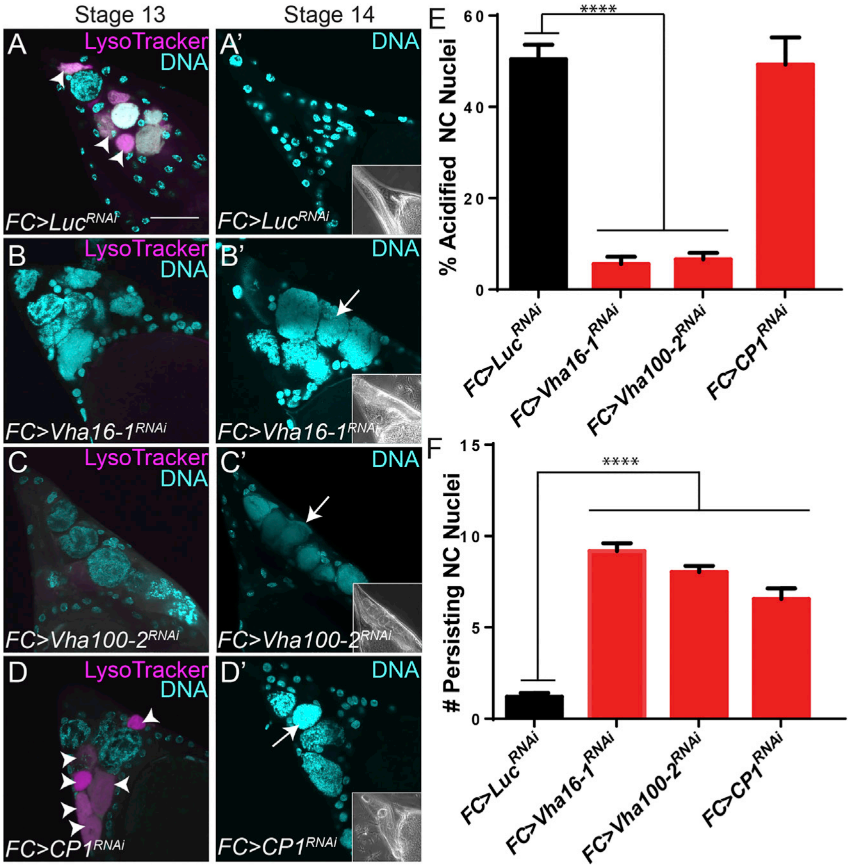

Figure 2. V-ATPases and Cathepsin L (CP1) Are Required Non-autonomously for NC Acidification and Clearance

(A–D') Stage 13 and 14 egg chambers labeled with LT (magenta) and DAPI (cyan).

(A and A') Control FC > *Luc*^{RNAi} stage 13 egg chamber has seven acidified NCs (arrowheads). All NCs are eliminated by stage 14. Phase-contrast insets show fully formed dorsal appendages in stage 14 egg chambers.

(B–C') FC knockdowns of V-ATPase subunits *Vha16-1*^{RNAi} and *Vha100-2*^{RNAi} have decreased NC acidification in stage 13 egg chambers (B and C) and persisting nuclei in stage 14 egg chambers (B' and C', arrows).

(D and D') FC > *CP1*^{RNAi} stage 13 egg chamber has six acidified NCs (arrowheads) and persisting nuclei in stage 14 egg chambers (arrow). Scale bars, 50 μm.

(E) Quantification of acidification of NCs in stage 13 egg chambers.

(F) Quantification of persisting NC nuclei remaining in stage 14 egg chambers (from 15 NCs per egg chamber). ****$p \leq 0.0001$

previously shown to correlate with Vha68-2 expression (Zhang et al., 2015). We colabeled egg chambers with an antibody against Eya that is expressed specifically in SFCs (Grammont, 2007). In stage 13 egg chambers, we found that Vha68-2 expression was increased 2.5-fold in SFCs (Figures 3A, 3A', S2A, and S2B) compared to other FCs. Colabeling with LT revealed that Vha68-2 expression was enriched in FCs adjacent to NCs (Figures 3B and 3B'). These findings demonstrate that V-ATPase expression is enriched in SFCs during stage 13 when NCs are being acidified.

To determine V-ATPase subcellular localization, we examined GFP protein traps for VhaSFD (subunit H), Vha13 (subunit G), and Vha44 (subunit C) (Buszczak et al., 2007; Morin et al., 2001; Nagarkar-Jaiswal et al., 2015). Null mutants of VhaSFD are homozygous lethal (Allan et al., 2005), but VhaSFD-GFP flies are homozygous viable, suggesting that the subunit functions normally with the GFP tag. All three of these V-ATPase protein traps had similar enrichment in SFCs, such as the Vha68-2 enhancer trap, but surprisingly, they localized to the plasma membrane of the SFCs (Figures 3C, 3C', S2C–S2E', and S3A–S3G''') rather than lysosomes (Figures S1A–S1G'). Immunohistochemistry with an antibody against ATP6V1B1, the human homolog of Vha55 (subunit B), also demonstrated an enrichment at the SFC plasma membrane (Figures 3D and 3D'), and colocalization was observed with a membrane marker (Figure S3). Taken together, these data demonstrate that V-ATPases are enriched in SFCs and localize to the plasma membrane. The localization of V-ATPases is different than either LT or LAMP1 staining (Figures 1 and S1A–S1G'), suggesting that V-ATPases are not delivered to the membrane by lysosome fusion. This localization combined with their requirement for NC acidification suggests that they function by extracellularly acidifying the nearby NCs, similar to V-ATPases acting at the plasma membrane in bone resorption or cancer cell invasion.

In human osteoclasts, V-ATPases are the primary proton pumps driving acidification of bone; however, to prevent a large difference in membrane potential, a chloride pump is also present at the plasma membrane (DiCiccio and Steinberg, 2011). Loss of the chloride channel (CLCN7) in humans leads to inefficient acidification of bone and leads to osteoporosis (Kornak et al., 2001). The *Drosophila* ortholog of CLCN7 is ClC-b, and it has been previously studied for its role in endolysosomes (Wong et al., 2017). To determine the expression and localization of ClC-b, we utilized a GFP protein trap. In stage 13 egg chambers, we found that ClC-b was enriched specifically in the SFCs and localized to the membrane as the NCs were becoming acidified (Figures 3E and 3E'). Altogether, this suggests that the SFCs may be utilizing the same machinery as osteoclasts for extracellular acidification.

V-ATPases and Cathepsins Are Non-autonomously Required for DNA Fragmentation of Nurse Cells

Previously, we found that disruption of phagocytosis genes in SFCs led to a block in both NC acidification and DNA fragmentation visualized by TUNEL staining (Timmons et al., 2016). To explore the role of acidification in DNA fragmentation, we performed TUNEL staining on V-ATPase and CP1 knockdowns. In

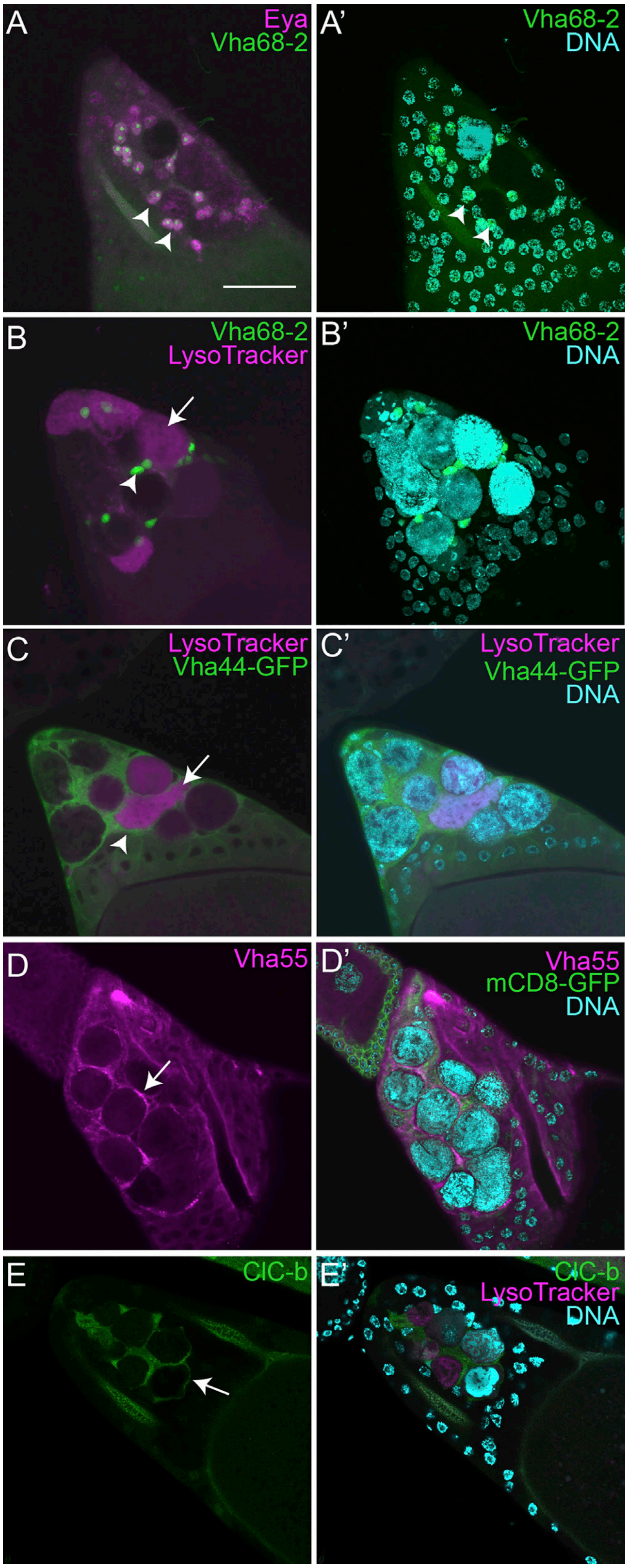

Figure 3. V-ATPases Are Enriched in Stretch Follicle Cells and Localize to the Plasma Membrane

(A and A') Z-projection of *Vha68-2-GFP* enhancer trap (green, arrowhead) stage 13 egg chamber labeled with anti-Eya (magenta). Scale bar, 50 μm.

(B and B') Z-projection of *Vha68-2* enhancer trap (green, arrowhead) stage 13 egg chamber labeled with DAPI (cyan) and LT (magenta, arrow).

(C and C') *Vha44-GFP* (green) stage 12 egg chamber labeled with LT (magenta) and DAPI (cyan).

(D and D') FC > *mCD8-GFP* (green) stage 13 egg chamber stained with anti-Vha55 (magenta) and DAPI (cyan).

(E and E') *ClC-b-GFP* (green) stage 13 egg chamber labeled with LT (magenta) and DAPI (cyan).

control stage 13 egg chambers, 60.9% of NCs labeled positively with TUNEL. Knocking down V-ATPase subunits or CP1 significantly reduced TUNEL-positive NCs to 8.56% (Vha16-1), 18.15% (Vha100-2), and 22.24% (CP1) (Figures 4A–4E). Thus, we conclude that V-ATPase activity and CP1 are both required for DNA fragmentation, a defining step of cell death (Galluzzi et al., 2015).

To visualize FC-derived CP1 during NC death, we expressed a hemagglutinin (HA)-tagged CP1 and a membrane-tethered GFP in the FCs. Initially, CP1 formed aggregates within the FCs; however, we also discovered that CP1 was released from the FCs into some of the NCs in stage 13 (Figures 4F–4F'''). Cathepsins are active only in acidic conditions, so we examined whether the NCs containing FC-derived CP1 were acidified. Consistently, acidified NCs contained CP1 that had been released from the FCs, whereas NCs that had not yet been acidified did not contain CP1 (Figures 4G–4G''').

To determine whether cathepsins were released from FCs by exocytosis, we knocked down two SNARE proteins associated with exocytosis, namely, Snap24 and Syx6 (Littleton, 2000; Niemeyer and Schwarz, 2000). We detected CP1 with an antibody that localized similarly to the CP1-HA construct (Figures S4A–S4A''). Quantification of the amount of CP1 localized in NCs revealed a significant decrease when Snap24 or Syx6 was knocked down in the FCs (Figures S4B–S4E). Additionally, knock down of either of these genes resulted in a significant decrease in NC acidification in stage 13 egg chambers and showed persisting NC nuclei in stage 14 egg chambers (Figures S4F and S4G). However, we found that a V-ATPase subunit localized to the SFC membrane normally in a Snap24 knockdown (Figure S4H), indicating that V-ATPases are targeted to the membrane independent of exocytosis.

Altogether, our findings suggest that CP1 is released from the FCs following NC acidification and both the acidification by V-ATPases and proteolytic activity of CP1 are required for the DNA fragmentation and elimination of the NCs.

DISCUSSION

Phagoptosis is defined as a type of cell death that requires phagocytosis machinery (Brown and Neher, 2012). We have previously demonstrated that NC death requires phagocytic machinery, such as Draper and Ced-12 (Santoso et al., 2018; Timmons et al., 2016). In the present study, we identified lysosome-associated genes required by the SFCs that non-autonomously control the elimination of NCs. To our knowledge, this

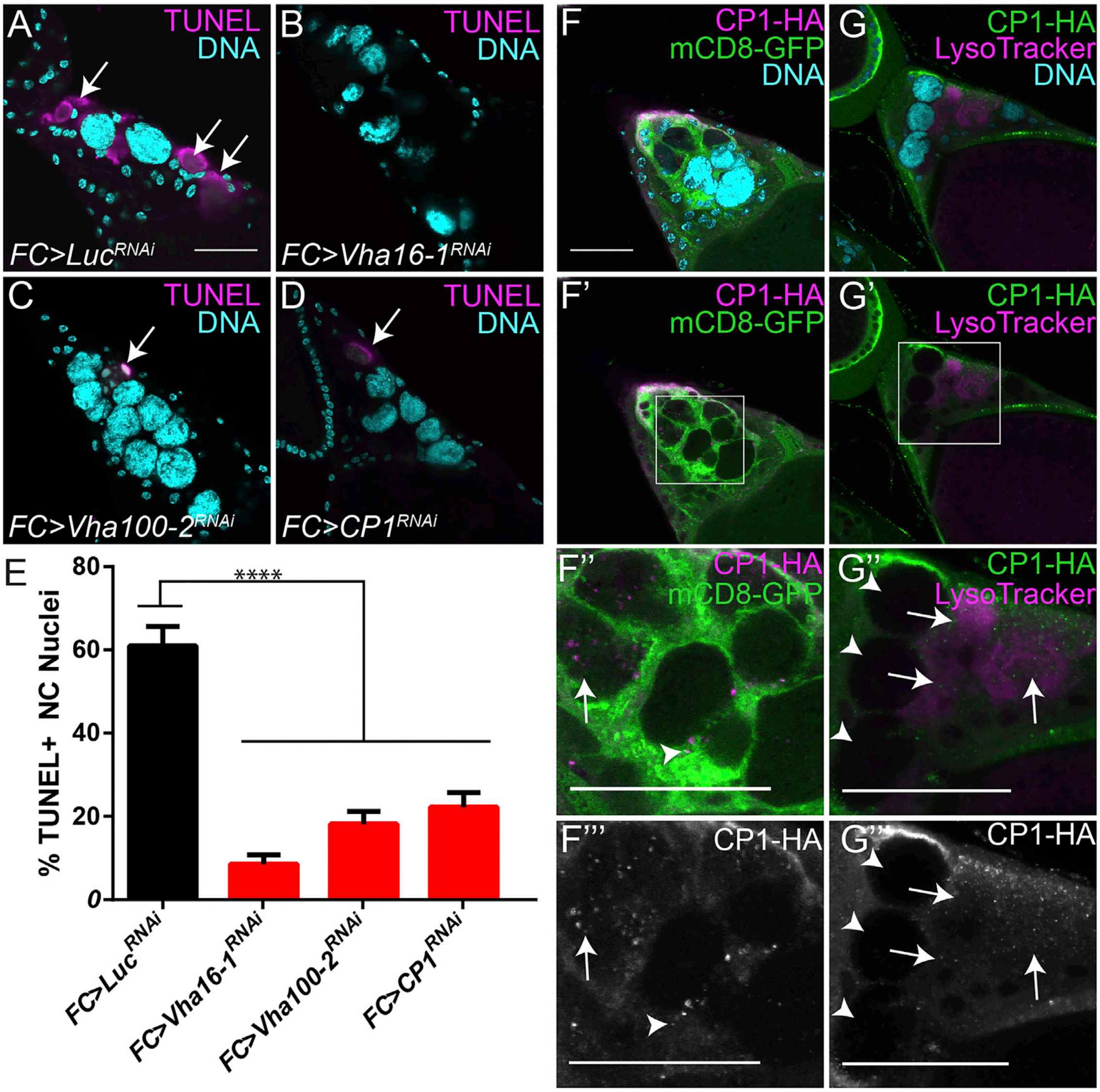

Figure 4. Cathepsin L Is Released by SFCs and Required for NC DNA Degradation

(A–D) Stage 13 egg chambers of the indicated genotypes labeled with TUNEL (magenta, arrows) and DAPI (cyan).

(E) Quantification of TUNEL-positive NC nuclei in stage 13 egg chambers. ****$p \leq 0.0001$.

(F–F''') *FC > mCD8-GFP, CP1-HA* stage 13 egg chamber labeled with anti-HA (magenta) and DAPI (cyan). CP1-HA (magenta) is produced in the FCs (green, arrowhead) and released into the NCs (arrow).

(G–G''') *FC>CP1-HA* egg chambers labeled with LT (magenta) and DAPI (cyan). CP1 is detected within LT-positive NCs (arrows) and not LT-negative NCs (arrowhead). Scale bars, 50 μm.

is the first example of V-ATPases at the plasma membrane driving acidification and subsequent cathepsin release to destroy a nearby cell. These findings suggest that signaling from the phagocytic machinery promotes this use of lysosomal proteins in NC elimination. Whether other examples of phagoptosis use the lysosomal machinery in this way remains to be determined.

V-ATPases are enriched at the plasma membrane of several specialized cell types in humans, such as osteoclasts, intercalated cells, clear cells, and some cancer cells. In insects, V-ATPases can be found on the plasma membranes of cells in certain tissues, such as Malpighian tubules (Bertram and Wessing, 1994; Day et al., 2008) and vas deferens (Bebas et al., 2002) to regulate pH or in earlier stages of oogenesis to play a role in bioelectric patterning (Krüger and Bohrmann, 2015). The specific isoforms associated with the plasma membrane V-ATPase holoenzyme have previously been identified (Allan et al., 2005). In this paper, we demonstrated that 7 of these plasma-membrane-associated V-ATPase subunits (Vha16-1, Vha100-2, Vha68-2, VhaSFD, Vha13, Vha44, and Vha55) are either enriched in the SFCs, localize to the SFC membrane, or are required for NC acidification.

Our previous work also highlighted Draper as being required for both the presence of LT vesicles in SFCs and NC acidification, suggesting that Draper initiates this process (Timmons et al., 2016). Other studies have demonstrated a link between Draper and autophagy genes (Etchegaray et al., 2016; McPhee and Baehrecke, 2010). Further work will need to be done to elucidate the upstream signaling components required to promote the phagoptotic potential of V-ATPases and cathepsins, and the role of SFC LT vesicles preceding NC acidification.

The findings reported here suggest a role for membrane-localized V-ATPases and cathepsin release in promoting cell death by phagoptosis. We have demonstrated that this mechanism is used during developmental NC death, but this mechanism may be used more broadly in other cell deaths that have been found to be non-apoptotic. Developmental germ cell death occurs in many organisms ranging from Hydra (Baum et al., 2005) to humans (Baker, 1963), and it is possible that surrounding somatic cells could contribute to the death of the germ cells in these other species. Our work also brings up the intriguing possibility that the lysosomal machinery can be harnessed to murder neighboring cells in other contexts.

STAR★METHODS

Detailed methods are provided in the online version of this paper and include the following:

- KEY RESOURCES TABLE
- CONTACT FOR REAGENT AND RESOURCE SHARING
- EXPERIMENTAL MODEL AND SUBJECT DETAILS
  - Fly Strains
  - Fly Husbandry
- METHODS DETAILS
  - Molecular Cloning
  - Live Imaging
  - Immunohistochemistry
  - TUNEL
  - Quantification and Statistical Analysis
  - Sample Sizes

SUPPLEMENTAL INFORMATION

Supplemental Information can be found online at https://doi.org/10.1016/j.celrep.2019.03.034.

ACKNOWLEDGMENTS

We thank our funding sources: the NIH (F31 GM115177 to A.A.M. and R01 GM060574 and R35 GM127338 to K.M.) and AAUW (to A.Y.). A.J.O. was supported by NSF-REU BIO-1659605 and Y.Z. was supported by Boston University UROP. W.-S.C. is supported by the National Research Foundation of Korea (NRF) grant funded by the Korean government (MSIP; NRF-2016M3C7A1905391, NRF-2016R1C1B3006969, and NRF-2018R1A4A1020922). We thank our colleagues for providing reagents and Michael Forgac and members of the lab for helpful conversations.

AUTHOR CONTRIBUTIONS

Conceptualization, A.A.M., A.Y., J.E., and K.M.; Methodology, A.A.M., A.Y., J.E., W.C., and K.M.; Investigation, A.A.M., A.J.O., Y.Z., and O.N.; Writing – Original Draft, A.A.M.; Writing – Review and Editing, A.A.M. and K.M.; Funding Acquisition, A.A.M., K.M., A.Y., and W.-S.C.; Supervision, A.A.M. and K.M.

DECLARATION OF INTERESTS

The authors declare no competing interests.

Received: April 20, 2018
Revised: February 18, 2019
Accepted: March 8, 2019
Published: April 2, 2019

REFERENCES

Allan, A.K., Du, J., Davies, S.A., and Dow, J.A.T. (2005). Genome-wide survey of V-ATPase genes in *Drosophila* reveals a conserved renal phenotype for lethal alleles. Physiol. Genomics *22*, 128–138.

Baker, T.G. (1963). A quantative and cytological study of germ cells in human ovaries. Proc. R. Soc. Lond. B Biol. Sci. *158*, 417–433.

Barcia, C., Ros, C.M., Annese, V., Carrillo-de Sauvage, M.A., Ros-Bernal, F., Gómez, A., Yuste, J.E., Campuzano, C.M., de Pablos, V., Fernandez-Villalba, E., and Herrero, M.T. (2012). ROCK/Cdc42-mediated microglial motility and gliapse formation lead to phagocytosis of degenerating dopaminergic neurons in vivo. Sci. Rep. *2*, 809.

Baron, R., Neff, L., Brown, W., Courtoy, P.J., Louvard, D., and Farquhar, M.G. (1988). Polarized secretion of lysosomal enzymes: co-distribution of cation-independent mannose-6-phosphate receptors and lysosomal enzymes along the osteoclast exocytic pathway. J. Cell Biol. *106*, 1863–1872.

Bass, B.P., Tanner, E.A., Mateos San Martín, D., Blute, T., Kinser, R.D., Dolph, P.J., and McCall, K. (2009). Cell-autonomous requirement for DNaseII in non-apoptotic cell death. Cell Death Differ. *16*, 1362–1371.

Baum, J.S., St George, J.P., and McCall, K. (2005). Programmed cell death in the germline. Semin. Cell Dev. Biol. *16*, 245–259.

Bebas, P., Cymborowski, B., and Giebultowicz, J.M. (2002). Circadian rhythm of acidification in insect vas deferens regulated by rhythmic expression of vacuolar H(+)-ATPase. J. Exp. Biol. *205*, 37–44.

Bertram, G., and Wessing, A. (1994). Intracellular pH regulation by the plasma membrane V-ATPase in Malpighian tubules of *Drosophila* larvae. J. Comp. Physiol. B *164*, 238–246.

Bischof, J., Björklund, M., Furger, E., Schertel, C., Taipale, J., and Basler, K. (2013). A versatile platform for creating a comprehensive UAS-ORFeome library in *Drosophila*. Development *140*, 2434–2442.

Brown, G.C., and Neher, J.J. (2012). Eaten alive! Cell death by primary phagocytosis: ‘phagoptosis’. Trends Biochem. Sci. *37*, 325–332.

Brown, G.C., and Neher, J.J. (2014). Microglial phagocytosis of live neurons. Nat. Rev. Neurosci. *15*, 209–216.

Brown, D., Paunescu, T.G., Breton, S., and Marshansky, V. (2009). Regulation of the V-ATPase in kidney epithelial cells: dual role in acid-base homeostasis and vesicle trafficking. J. Exp. Biol. *212*, 1762–1772.

Buszczak, M., Paterno, S., Lighthouse, D., Bachman, J., Planck, J., Owen, S., Skora, A.D., Nystul, T.G., Ohlstein, B., Allen, A., et al. (2007). The carnegie protein trap library: a versatile tool for *Drosophila* developmental studies. Genetics *175*, 1505–1531.

Cooley, L., Verheyen, E., and Ayers, K. (1992). chickadee encodes a profilin required for intercellular cytoplasm transport during *Drosophila* oogenesis. Cell *69*, 173–184.

Cotter, K., Stransky, L., McGuire, C., and Forgac, M. (2015). Recent Insights into the Structure, Regulation, and Function of the V-ATPases. Trends Biochem. Sci. *40*, 611–622.

Day, J.P., Wan, S., Allan, A.K., Kean, L., Davies, S.A., Gray, J.V., and Dow, J.A.T. (2008). Identification of two partners from the bacterial Kef exchanger family for the apical plasma membrane V-ATPase of Metazoa. J. Cell Sci. *121*, 2612–2619.

DiCiccio, J.E., and Steinberg, B.E. (2011). Lysosomal pH and analysis of the counter ion pathways that support acidification. J. Gen. Physiol. *137*, 385–390.

Dinkins, M.B., Fratto, V.M., and Lemosy, E.K. (2008). Integrin alpha chains exhibit distinct temporal and spatial localization patterns in epithelial cells of the *Drosophila* ovary. Dev. Dyn. *237*, 3927–3939.

Duhart, J.C., Parsons, T.T., and Raftery, L.A. (2017). The repertoire of epithelial morphogenesis on display: Progressive elaboration of *Drosophila* egg structure. Mech. Dev. *148*, 18–39.

Etchegaray, J.I., Elguero, E.J., Tran, J.A., Sinatra, V., Feany, M.B., and McCall, K. (2016). Defective Phagocytic Corpse Processing Results in Neurodegeneration and Can Be Rescued by TORC1 Activation. J. Neurosci. *36*, 3170–3183.

Fishilevich, E., Fitzpatrick, J.A.J., and Minden, J.S. (2010). pHMA, a pH-sensitive GFP reporter for cell engulfment, in *Drosophila* embryos, tissues, and cells. Dev. Dyn. *239*, 559–573.

Foley, K., and Cooley, L. (1998). Apoptosis in late stage *Drosophila* nurse cells does not require genes within the H99 deficiency. Development *125*, 1075–1082.

Galluzzi, L., Bravo-San Pedro, J.M., Vitale, I., Aaronson, S.A., Abrams, J.M., Adam, D., Alnemri, E.S., Altucci, L., Andrews, D., Annicchiarico-Petruzzelli, M., et al. (2015). Essential versus accessory aspects of cell death: recommendations of the NCCD 2015. Cell Death Differ. *22*, 58–73.

Goentoro, L.A., Yakoby, N., Goodhouse, J., Schüpbach, T., and Shvartsman, S.Y. (2006). Quantitative analysis of the GAL4/UAS system in *Drosophila* oogenesis. Genesis *44*, 66–74.

Grammont, M. (2007). Adherens junction remodeling by the Notch pathway in *Drosophila melanogaster* oogenesis. J. Cell Biol. *177*, 139–150.

Guild, G.M., Connelly, P.S., Shaw, M.K., and Tilney, L.G. (1997). Actin filament cables in *Drosophila* nurse cells are composed of modules that slide passively past one another during dumping. J. Cell Biol. *138*, 783–797.

Hancock, J.F., Cadwallader, K., Paterson, H., and Marshall, C.J. (1991). A CAAX or a CAAL motif and a second signal are sufficient for plasma membrane targeting of ras proteins. EMBO J. *10*, 4033–4039.

Jacobson, M.D., Weil, M., and Raff, M.C. (1997). Programmed cell death in animal development. Cell *88*, 347–354.

King, R.C. (1970). Ovarian Development in *Drosophila melanogaster* (Academic Press).

Kornak, U., Kasper, D., Bösl, M.R., Kaiser, E., Schweizer, M., Schulz, A., Friedrich, W., Delling, G., and Jentsch, T.J. (2001). Loss of the ClC-7 chloride channel leads to osteopetrosis in mice and man. Cell *104*, 205–215.

Krüger, J., and Bohrmann, J. (2015). Bioelectric patterning during oogenesis: stage-specific distribution of membrane potentials, intracellular pH and ion-transport mechanisms in *Drosophila* ovarian follicles. BMC Dev. Biol. *15*, 1.

Kutscher, L.M., and Shaham, S. (2017). Non-apoptotic cell death in animal development. Cell Death Differ. *24*, 1326–1336.

Lana, D., Ugolini, F., Melani, A., Nosi, D., Pedata, F., and Giovannini, M.G. (2017). The neuron-astrocyte-microglia triad in CA3 after chronic cerebral hypoperfusion in the rat: Protective effect of dipyridamole. Exp. Gerontol. *96*, 46–62.

Littleton, J.T. (2000). A genomic analysis of membrane trafficking and neurotransmitter release in Drosophila. J. Cell Biol. *150*, F77–F82.

Liu, G., Fiala, M., Mizwicki, M.T., Sayre, J., Magpantay, L., Siani, A., Mahanian, M., Chattopadhyay, M., La Cava, A., and Wiedau-Pazos, M. (2012). Neuronal phagocytosis by inflammatory macrophages in ALS spinal cord: inhibition of inflammation by resolvin D1. Am. J. Neurodegener. Dis. *1*, 60–74.

Lübke, T., Lobel, P., and Sleat, D.E. (2009). Proteomics of the lysosome. Biochim. Biophys. Acta *1793*, 625–635.

Martins, I., Raza, S.Q., Voisin, L., Dakhli, H., Law, F., De Jong, D., Allouch, A., Thoreau, M., Brenner, C., Deutsch, E., and Perfettini, J.L. (2017). Entosis: The emerging face of non-cell-autonomous type IV programmed death. Biomed. J. *40*, 133–140.

McPhee, C.K., and Baehrecke, E.H. (2010). The engulfment receptor Draper is required for autophagy during cell death. Autophagy *6*, 1192–1193.

Morin, X., Daneman, R., Zavortink, M., and Chia, W. (2001). A protein trap strategy to detect GFP-tagged proteins expressed from their endogenous loci in *Drosophila*. Proc. Natl. Acad. Sci. USA *98*, 15050–15055.

Nagarkar-Jaiswal, S., DeLuca, S.Z., Lee, P.T., Lin, W.W., Pan, H., Zuo, Z., Lv, J., Spradling, A.C., and Bellen, H.J. (2015). A genetic toolkit for tagging intronic MiMIC containing genes. eLife *4*, 2–9.

Neher, J.J., Emmrich, J.V., Fricker, M., Mander, P.K., Théry, C., and Brown, G.C. (2013). Phagocytosis executes delayed neuronal death after focal brain ischemia. Proc. Natl. Acad. Sci. USA *110*, E4098–E4107.

Niemeyer, B.A., and Schwarz, T.L. (2000). SNAP-24, a *Drosophila* SNAP-25 homologue on granule membranes, is a putative mediator of secretion and granule-granule fusion in salivary glands. J. Cell Sci. *113*, 4055–4064.

Overholtzer, M., Mailleux, A.A., Mouneimne, G., Normand, G., Schnitt, S.J., King, R.W., Cibas, E.S., and Brugge, J.S. (2007). A nonapoptotic cell death process, entosis, that occurs by cell-in-cell invasion. Cell *131*, 966–979.

Peters, N.C., and Berg, C.A. (2016). In vitro culturing and live imaging of *Drosophila* egg chambers: A history and adaptable method. Methods Mol. Biol. *1457*, 35–68.

Peterson, J.S., and McCall, K. (2013). Combined inhibition of autophagy and caspases fails to prevent developmental nurse cell death in the *Drosophila melanogaster* ovary. PLoS One *8*, e76046.

Peterson, J.S., Timmons, A.K., Mondragon, A.A., and McCall, K. (2015). The end of the beginning: Cell death in the Germline. Curr. Top. Dev. Biol. *114*, 93–119.

Pfeiffer, B.D., Truman, J.W., and Rubin, G.M. (2012). Using translational enhancers to increase transgene expression in *Drosophila*. Proc. Natl. Acad. Sci. USA *109*, 6626–6631.

Qin, A., Cheng, T.S., Pavlos, N.J., Lin, Z., Dai, K.R., and Zheng, M.H. (2012). V-ATPases in osteoclasts: structure, function and potential inhibitors of bone resorption. Int. J. Biochem. Cell Biol. *44*, 1422–1435.

Rozhin, J., Sameni, M., Ziegler, G., and Sloane, B.F. (1994). Pericellular pH affects distribution and secretion of cathepsin B in malignant cells. Cancer Res. *54*, 6517–6525.

Santoso, C.S., Meehan, T.L., Peterson, J.S., Cedano, T.M., Turlo, C.V., and McCall, K. (2018). The ABC Transporter Eato Promotes Cell Clearance in the *Drosophila melanogaster*. Ovary. G3 *8*, 833–843.

Settembre, C., Fraldi, A., Medina, D.L., and Ballabio, A. (2013). Signals from the lysosome: a control centre for cellular clearance and energy metabolism. Nat. Rev. Mol. Cell Biol. *14*, 283–296.

Shum, W.W.C., Da Silva, N., Brown, D., and Breton, S. (2009). Regulation of luminal acidification in the male reproductive tract via cell-cell crosstalk. J. Exp. Biol. *212*, 1753–1761.

Spradling, A. (1993). Developmental genetics of oogenesis. In The Development of Drosophila Melanogaster, M. Bate and A. Martinez-Arias, eds. (Cold Spring Harbor Lab Press), pp. 1–70.

Stransky, L., Cotter, K., and Forgac, M. (2016). The Function of V-ATPases in Cancer. Physiol. Rev. *96*, 1071–1091.

Tantama, M., Hung, Y.P., and Yellen, G. (2011). Imaging intracellular pH in live cells with a genetically encoded red fluorescent protein sensor. J. Am. Chem. Soc. *133*, 10034–10037.

Tilly, J.L. (2001). Commuting the death sentence: how oocytes strive to survive. Nat. Rev. Mol. Cell Biol. *2*, 838–848.

Timmons, A.K., Meehan, T.L., Gartmond, T.D., and McCall, K. (2013). Use of necrotic markers in the *Drosophila* ovary. Methods Mol. Biol. *1004*, 215–228.

Timmons, A.K., Mondragon, A.A., Schenkel, C.E., Yalonetskaya, A., Taylor, J.D., Moynihan, K.E., Etchegaray, J.I., Meehan, T.L., and McCall, K. (2016). Phagocytosis genes nonautonomously promote developmental cell death in the *Drosophila* ovary. Proc. Natl. Acad. Sci. USA *113*, E1246–E1255.

Timmons, A.K., Mondragon, A.A., Meehan, T.L., and McCall, K. (2017). Control of non-apoptotic nurse cell death by engulfment genes in *Drosophila*. Fly (Austin) *11*, 104–111.

Tryselius, Y., and Hultmark, D. (1997). Cysteine proteinase 1 (CP1), a cathepsin L-like enzyme expressed in the *Drosophila melanogaster* haemocyte cell line mbn-2. Insect Mol. Biol. *6*, 173–181.

Wong, C.O., Gregory, S., Hu, H., Chao, Y., Sepúlveda, V.E., He, Y., Li-Kroeger, D., Goldman, W.E., Bellen, H.J., and Venkatachalam, K. (2017). Lysosomal Degradation Is Required for Sustained Phagocytosis of Bacteria by Macrophages. Cell Host Microbe *21*, 719–730.e6.

Zabel, M.K., Zhao, L., Zhang, Y., Gonzalez, S.R., Ma, W., Wang, X., Fariss, R.N., and Wong, W.T. (2016). Microglial phagocytosis and activation underlying photoreceptor degeneration is regulated by CX3CL1-CX3CR1 signaling in a mouse model of retinitis pigmentosa. Glia *64*, 1479–1491.

Zhang, T., Zhou, Q., Ogmundsdottir, M.H., Möller, K., Siddaway, R., Larue, L., Hsing, M., Kong, S.W., Goding, C.R., Palsson, A., et al. (2015). Mitf is a master regulator of the v-ATPase, forming a control module for cellular homeostasis with v-ATPase and TORC1. J. Cell Sci. *128*, 2938–2950.

Zhao, L., Zabel, M.K., Wang, X., Ma, W., Shah, P., Fariss, R.N., Qian, H., Parkhurst, C.N., Gan, W.-B., and Wong, W.T. (2015). Microglial phagocytosis of living photoreceptors contributes to inherited retinal degeneration. EMBO Mol. Med. *7*, 1179–1197.

STAR★METHODS

KEY RESOURCES TABLE

REAGENT or RESOURCE	SOURCE	IDENTIFIER
Antibodies		
Eya	DSHB	Cat# Eya10H6; RRID:AB_528232
ATP6V1B1	Abgent	Cat# AP11538C-ev; RRID:AB_2797396
Goat-α-mouse Cy3	Jackson ImmunoResearch	Cat# 115-165-003; RRID:AB_2338680
CP1	R&D Systems	Cat# MAB22591; RRID:AB_2087830
LAMP1	Abcam	Cat# Ab30687; RRID:AB_775973
Goat-α-rabbit 647	Jackson ImmunoResearch	Cat# 111-605-144; RRID:AB_2338078
Bacterial and Virus Strains		
NEB 5′ 5-alpha F’Iq Competent *E. coli*	NEB	C2992H
Chemicals, Peptides, and Recombinant Proteins		
LysoTracker	Thermo Fisher Scientific	L75283
Vectashield with DAPI	Vector Laboratories	H-1200
Hoechst 33342	Thermo Fisher Scientific	62249
In Situ Cell Death Detection Kit, TMR Red	Sigma-Aldrich	12156792910
Experimental Models: Organisms/Strains		
(Stretch follicle cell) PG150-GAL4	Dr. Ellen LeMosy	N/A
(Follicle cell) GR1-GAL4	Dr. Trudi Schüpbach	N/A
(Germline) NGT;nanos-GAL4	Dr. Pernille Rorth	N/A
UAS-CP1-HA	FlyORF	780
Vha68-2-GFP	Dr. Francesca Pignoni	N/A
ClC-b-GFP	Dr. Kartik Venkatachalam	N/A
Vha13-GFP	Bloomington Stock Center	50828
Vha44-GFP	Bloomington Stock Center	63202
VhaSFD-GFP	Bloomington Stock Center	6840
Luciferase RNAi (JF01355)	Bloomington Stock Center	31603
Vha100-2 RNAi (HMC05732)	Bloomington Stock Center	64859
Vha16-1 RNAi (HMS02171)	Bloomington Stock Center	40923
CP1 RNAi (HMS00725)	Bloomington Stock Center	32932
Snap24 RNAi (JF03146)	Bloomington Stock Center	28719
Syx6 RNAi (JF03125)	Bloomington Stock Center	28505
Myr-GFP (pJFRC29- 10XUAS-IVS-myr::GFP-p10)	Dr. Norbert Perrimon	N/A
Myr-RFP	Bloomington Stock Center	7118
Gal80ts	Bloomington Stock Center	7019
Recombinant DNA		
pHRed-cAAX Plasmid	Dr. Won-Suk Chung	N/A
UASp (pPW) Plasmid	DGRC	1130

CONTACT FOR REAGENT AND RESOURCE SHARING

Any requests for resources and reagents should be directed to the lead contact, Kim McCall (kmccall@bu.edu).

EXPERIMENTAL MODEL AND SUBJECT DETAILS

Fly Strains

PG150-GAL4 was used to drive expression in SFCs (indicated by SFC > in figures) and was provided by Ellen LeMosy (Dinkins et al., 2008). *GR1-GAL4* was used to drive expression in FCs (indicated by FC > in figures) and was provided by Trudi Schüpbach (Goentoro et al., 2006). *Luciferase*^{RNAi} was used as a control for all RNAi experiments. *UAS-CP1-HA* was obtained from FlyORF (Bischof et al., 2013). *Vha68-2-GFP* was provided by Francesca Pignoni (Zhang et al., 2015). *ClC-b-GFP* was provided by Kartik Venkatachalam (Wong et al., 2017). *UAS-Myr-GFP* was provided by Norbert Perrimon (Pfeiffer et al., 2012).

Fly Husbandry

Flies were age matched and dissected before 2 weeks of age. All RNAi crosses were performed in combination with *Gal80*^{ts} at 18°C and moved to 29°C for 48 hours, except the crosses for Figure S4 which were done without *Gal80*^{ts} at 25°C and moved to 29°C for 48 hours. All other fly crosses were kept at 25°C. All flies were well fed with yeast paste for 2 days (new yeast paste each day) before dissection to increase egg chamber production.

METHODS DETAILS

Molecular Cloning

pHRed-CAAX was PCR amplified from a plasmid obtained from Dr. Won-Suk Chung and cloned into a Gateway entry vector pENTR using the pENTR/D-TOPO cloning kit (Invitrogen). Gateway LR Clonase (Invitrogen) was used to recombine the construct downstream of the GAL4 responsive promoter UASp (in the plasmid pPW, received from *Drosophila* Genome Resource Center, Bloomington, IN). The P element vector with pHRed-CAAX was injected into embryos for P-element transformation by BestGene (Chino Hills, CA).

Live Imaging

Live imaging was performed as described (Peters and Berg, 2016). Briefly, stage 13 egg chambers were individually dissected from flies in room temperature Schneider's medium. Once isolated, egg chambers were transferred to Schneider's medium (product number 21720-024, Thermo Fisher Scientific) with LysoTracker (LT, LysoTracker Red DND-99 – Invitrogen by Thermo Fisher Scientific L75283 – 1:1000 dilution) and Hoechst 33342 (product number 62249, Thermo Fisher Scientific– 10 μM). Egg chambers in solution were transferred to the imaging chamber which had a glass bottom. An immobilization blanket (small Kimwipe) was used to keep egg chambers in place during imaging. The immobilization blanket was placed in the solution on top of the egg chambers and a brass washer was placed on the blanket to hold it in place. Live imaging was captured on a Nikon C2+Si laser scanning confocal microscope.

Immunohistochemistry

For LT staining, whole ovaries were dissected from flies in Grace's insect media (product number BW04-457F, Thermo Fisher Scientific) and incubated in LT solution (Thermo Fisher Scientific, 1:50 in PBS) for 6 minutes, rinsed with PBS for 30 min while rotating, fixed in 300 uL Graces, 200 uL Heptane and 4% Paraformaldehyde, washed with PBS + 1% Triton X-100 (PBT) for 15 min, and stained with DAPI. For antibody staining, ovaries were immediately fixed after dissection, washed with PBT for 1 hour, blocked with PBANG (PBT, 0.5% BSA, 5% Normal Goat Serum), and incubated overnight in primary antibody diluted in PBANG. Samples were rinsed with PBT twice, washed with PBT + 0.5% BSA for 2 hours, and incubated in secondary antibody diluted in PBANG for 1 hour, protected from light. Samples were rinsed with PBT twice, washed with PBT + 0.5% BSA for 2 hours, rinsed with PBS, and incubated in 2 drops of Vectashield with DAPI overnight before mounting. Primary antibodies were Eya (Developmental Studies Hybridoma Bank), a stretch follicle cell specific marker, at 1:300, ATP6V1B1 (Abgent) at 1:10, CP1 (R&D Systems) at 1:200, LAMP1 (abcam) at 1:500. Secondary goat-α-mouse Cy3 (Jackson ImmunoResearch) was used 1:100 and goat-α-rabbit 647 (Jackson ImmunoResearch) was used at 1:100. Imaging of egg chambers was performed on an Olympus Fluoview FV10i laser scanning confocal or a Nikon C2+Si laser scanning confocal microscope.

TUNEL

To assay for DNA fragmentation, we dissected ovaries from flies in 2% paraformaldehyde in PBS with 0.1% Triton X-100. Ovaries were then fixed for 45 min, rinsed with PBT twice, washed in PBT for 30 min, permeabilized in PBS with 0.1% sodium citrate and 0.1% Triton X-100 at 65°C for 30 min. Tissue was then washed 3 times in PBT for 20 min each. Tissue was incubated in 36 uL TUNEL label solution and 4 uL enzyme solution (*In Situ* Cell Death Detection Kit, TMR Red – Sigma-Aldrich, Cat #12 156 792 910) for 3 hours at 37°C. Samples were washed 4X in PBS for a total time of 1 hour and mounted in Vectashield with DAPI.

Quantification and Statistical Analysis

All data were graphed and analyzed in Graphpad Prism, and an unpaired t test was performed on each set of data compared to control. The mean was graphed ± SEM. Each experiment had at least three biological replicates. At least 10 flies were randomly selected

for each replicate and egg chambers were dissected and staged using previously described methods (Spradling, 1993) on an Olympus BX60 upright fluorescence microscope. For quantification of Vha68-2 GFP intensity (Figure S2B), the mean GFP intensity of Eya^{+} follicle cells (SFCs) and Eya^{-} follicle cells was measured by ImageJ after outlining only Eya^{+} or Eya^{-} nuclei. Measurement of CP1 pixels (Figure S4E) was also performed in ImageJ. NC nuclei (regions of interest) were outlined based on DAPI staining, the CP1 channel was converted to a black and white image, and the pixels in the regions of interest were counted in the CP1 channel.

Sample Sizes

Figure 2E - *GR1* (FC) > Luc^{RNAi}, n = 72 egg chambers; *GR1* (FC) > $Vha16\text{-}1^{RNAi}$, n = 28; *GR1* (FC) > Vha100-2^{RNAi}, n = 50; *GR1* (FC) > $CP1^{RNAi}$, n = 19.

Figure 2F - *GR1* (FC) > Luc^{RNAi}, n = 85; *GR1* (FC) > $Vha16\text{-}1^{RNAi}$, n = 72; *GR1* (FC) > Vha100-2^{RNAi}, n = 118; *GR1* (FC) > $CP1^{RNAi}$, n = 38.

Figure 4E - *GR1* (FC) > Luc^{RNAi}, n = 42; *GR1* (FC) > $Vha16\text{-}1^{RNAi}$, n = 47; *GR1* (FC) > Vha100-2^{RNAi}, n = 43; *GR1* (FC) > $CP1^{RNAi}$, n = 50.

Cell Reports, Volume *27*

Supplemental Information

Lysosomal Machinery Drives Extracellular Acidification to Direct Non-apoptotic Cell Death

Albert A. Mondragon, Alla Yalonetskaya, Anthony J. Ortega, Yuanhang Zhang, Oandy Naranjo, Johnny Elguero, Won-Suk Chung, and Kimberly McCall

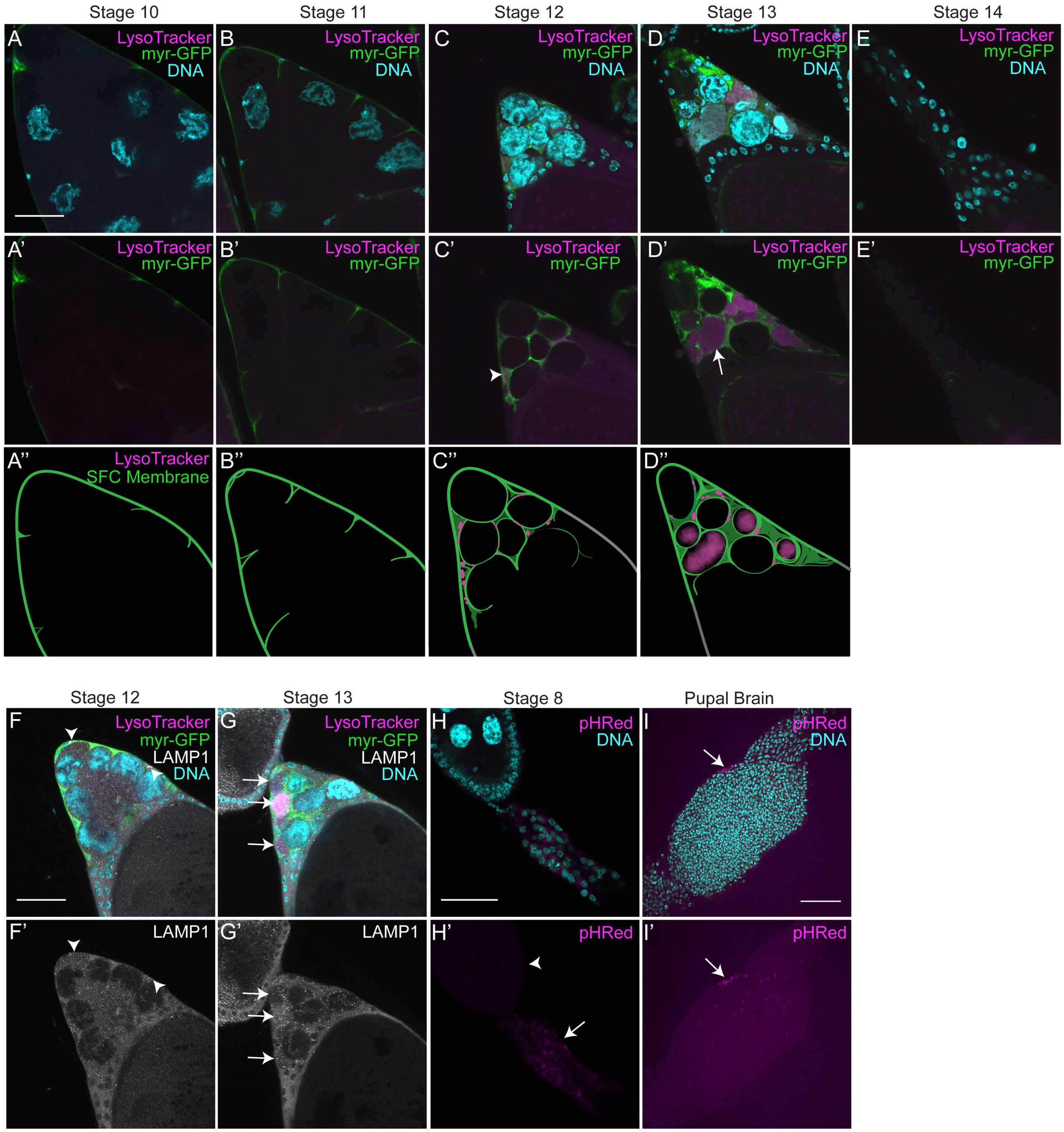

Supplemental Figure 1 – Analysis of pH sensitive markers, Related to Figure 1
(A-E') Comparison of LysoTracker staining and stretch follicle cell progression. *PG150(SFC)>myr-GFP* egg chambers labeled with LT (magenta). SFCs begin to surround NCs in stage 11. LT puncta accumulate around NCs within SFCs beginning at stage 12 (arrowhead). NCs become acidified at stage 13 (arrow). LT staining and SFC membranes are no longer detected in stage 14. **(A''-D'')** Schematic showing patterns of SFC membranes compared to LysoTracker. (**F-G'**) Comparison of LysoTracker with lysosomal marker LAMP-1. *PG150(SFC)>myr-GFP* egg chambers labeled with LT (magenta), LAMP-1 (White), and DAPI (cyan). (**H-I'**) Demonstration of pHRed as an engulfment marker. (**H-H'**) *NGT;nanos (germline)>pHRed* egg chambers labeled with DAPI (cyan). Healthy egg chamber (arrowhead) next to pHRed positive dying stage 8 egg chamber (arrow). (**I-I'**) *elav* (neuronal)>*pHRed* pupal brain labeled with DAPI (cyan). pHRed vesicles associate with regions of cell death in a pupal brain (arrows). Scale bars = 50 µm.

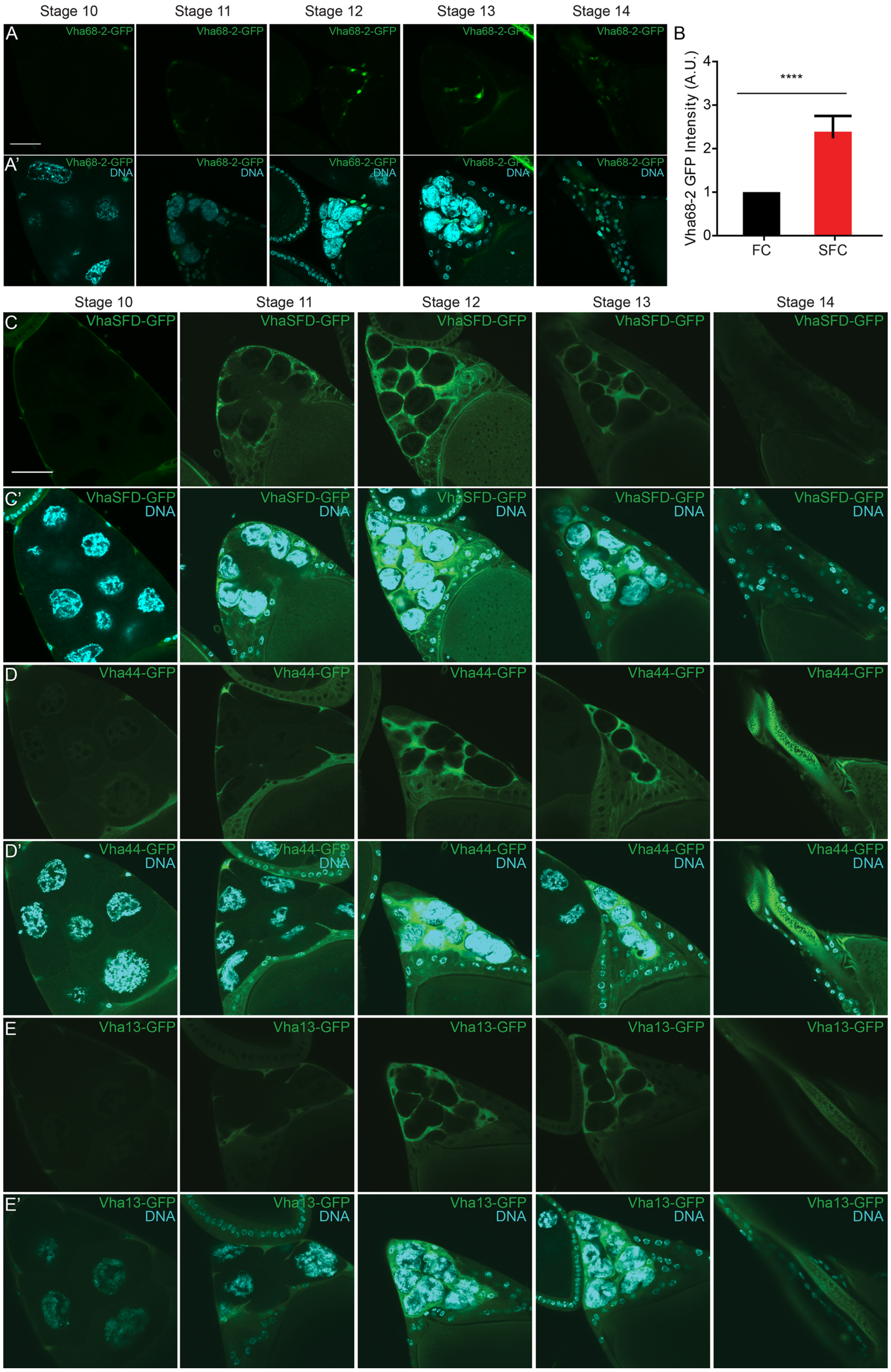

Supplemental Figure 2 – Vha68-2 enrichment in stretch follicle cells, and VhaSFD, Vha44, and Vha13 enrichment and localization in stretch follicle cells, Related to Figure 3
(A-A') *Vha68-2-GFP* enhancer trap (green) stage 10-14 egg chambers with DAPI (cyan). (**B**) Vha68-2 GFP intensity (arbitrary units) in follicle cells (n=33) and stretch follicle cells (n=29). Two-tailed students t-test, data are mean +/- SEM. **** $P \leq 0.0001$. Scale bar = 50 µm. (**C-C')** *VhaSFD-GFP* protein trap (green) stage 10-14 egg chambers with DAPI (cyan). (**D-D')** *Vha44-GFP* protein trap (green) stage 10-14 egg chambers with DAPI (cyan). (**E-E')** *Vha13-GFP* protein trap (green) stage 10-14 egg chambers with DAPI (cyan). Scale bar = 50 µm.

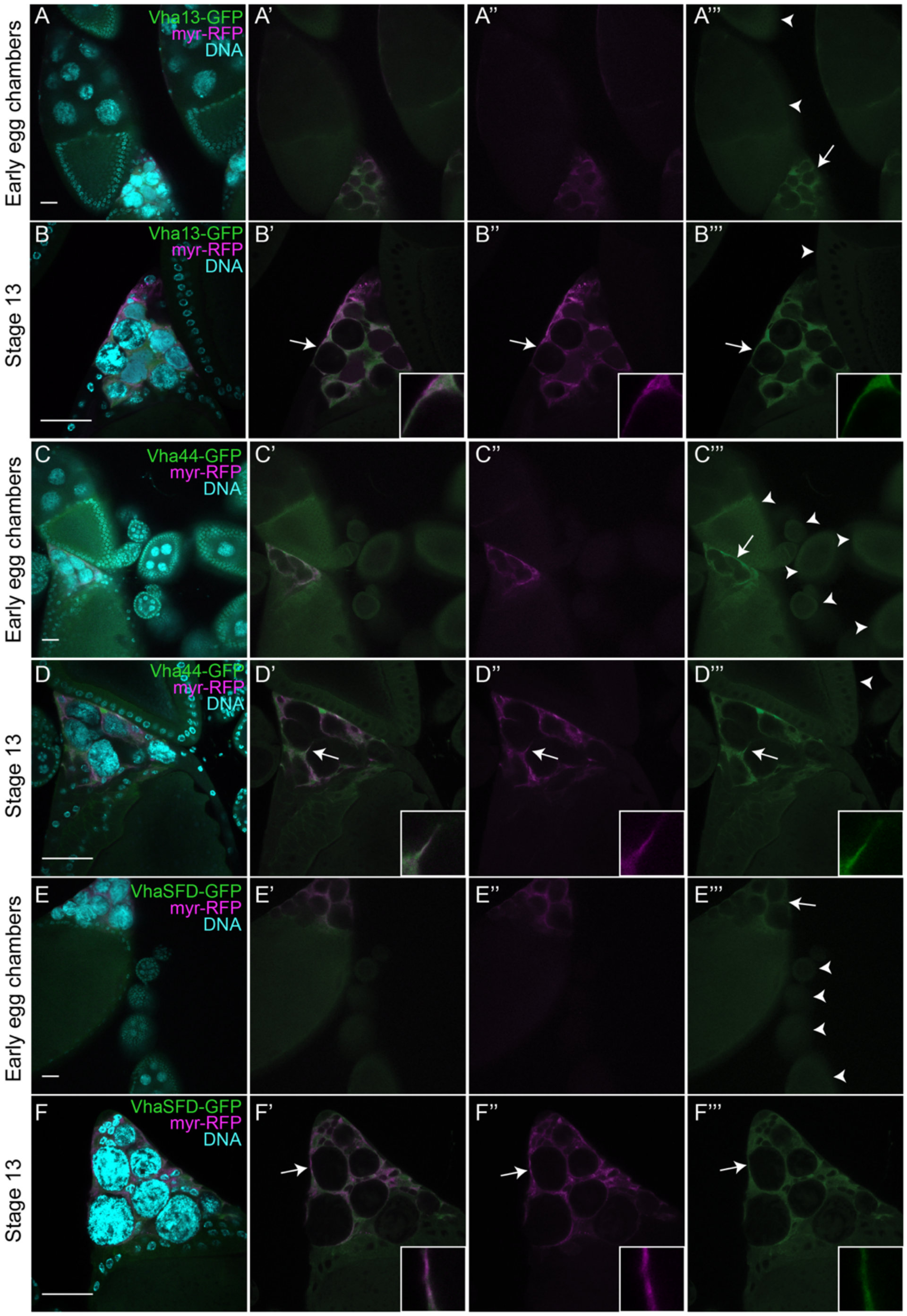

Supplemental Figure 3 – V-ATPases localize to the plasma membrane of stretch follicle cells, Related to Figure 3

(A-F''') Egg chambers expressing *PG150(SFC)>myr-RFP* (magenta) and the indicated V-ATPase subunit protein traps (green) stained with DAPI (cyan). (**A-A''', C-C''', E-E'''**) are lower magnification images with early stage egg chambers (arrowheads) compared to stage 13 egg chambers (arrow). The V-ATPase GFP protein traps are enriched in the SFCs of stage 13 egg chambers. (**B-B''', D-D''', F-F'''**) are higher magnification images of the stage 13 egg chambers. The V-ATPase GFP protein traps are localized to the stretch follicle cell plasma membrane (arrow). Insets show higher magnification of the overlap of V-ATPase GFP (green) and stretch follicle cell plasma membrane (magenta). Scale bars = 50 µm.

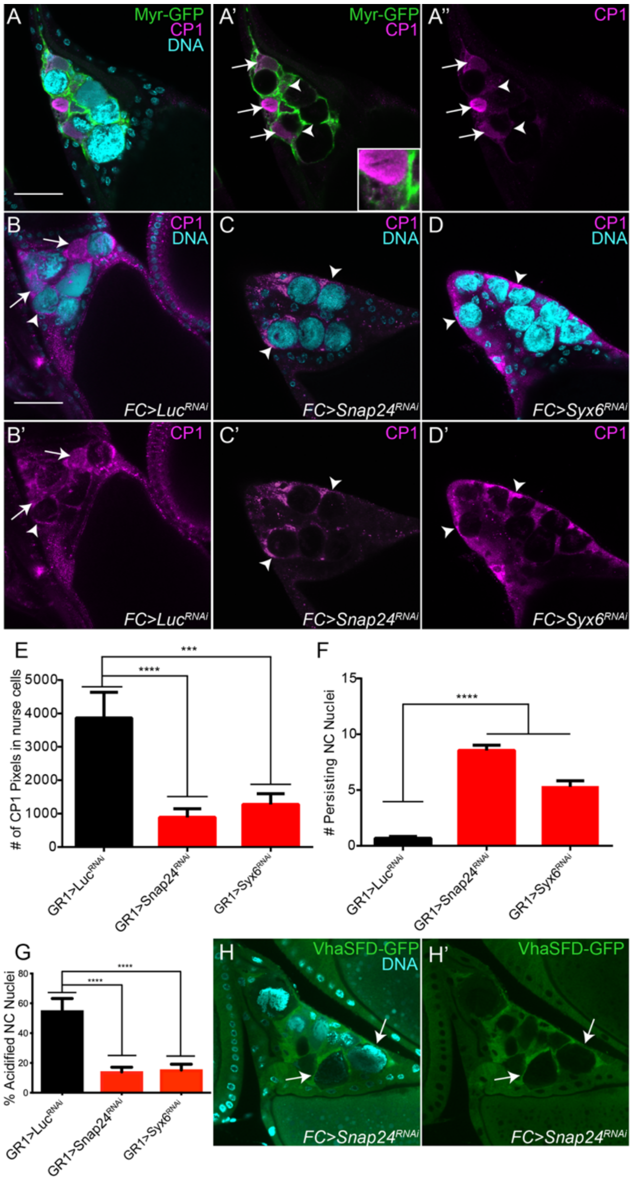

Supplemental Figure 4 – Snap24 and Syx6 are required for nurse cell removal and CP1 release, Related to Figure 4

(A-A'') *PG150(SFC)>myr-GFP* (green) stage 13 egg chamber labeled with anti-CP1 (magenta) and DAPI (cyan). CP1 is present in nurse cells (arrows) and stretch follicle cells (arrowheads). Inset shows CP1 localization inside nurse cell and stretch follicle cell. (**B-D'**) Stage 13 egg chambers from RNAi knockdowns labeled with anti-CP1 (magenta) and DAPI (cyan). In the control (B-B') CP1 surrounds some of the nurse cells (arrowheads) and is in other nurse cells (arrows). In the *Snap24* and *Syx6* knockdowns, CP1 is predominantly in the SFCs. **E,** Quantification of CP1 pixels which overlap nurse cell nuclei. The amount of CP1 in nurse cells is lower in the *Snap24* and *Syx6* knockdowns compared to the *Luc* control. Sample size: *GR1 (FC)>Luc^{RNAi}*, n = 20; *GR1(FC)>Snap24^{RNAi}*, n= 22; *GR1(FC)>Syx6^{RNAi}*, n = 24. Two-tailed students t-test, data are mean +/- SEM. **** P<0.0001 and *** P<0.005. (**F**) Quantification of persisting nurse cell nuclei in stage 14 egg chambers. Sample size: *GR1 (FC)>Luc^{RNAi}*, n = 84; *GR1(FC)>Snap24^{RNAi}*, n= 41; *GR1(FC)>Syx6^{RNAi}*, n = 51. Two-tailed students t-test, data are mean +/- SEM. **** P<0.0001. (**G**) Quantification of acidification of NCs in stage 13 egg chambers. Sample size: *GR1 (FC)>Luc^{RNAi}*, n = 10 ; *GR1(FC)>Snap24^{RNAi}*, n= 32 ; *GR1(FC)>Syx6^{RNAi}*, n = 22 . Two-tailed students t-test, data are mean +/- SEM. **** P<0.0001. (**H-H'**) *VhaSFD-GFP* protein trap (green, arrows) with RNAi knockdown of *Snap24* and labeled with DAPI (cyan). Scale bars = 50 μm.
